# Topical Diclofenac Solution for Osteoarthritis of the Knee: An Updated Meta-Analysis of Randomized Controlled Trials

**DOI:** 10.1155/2020/1758071

**Published:** 2020-11-24

**Authors:** Tao Ling, Jiao Jiao Li, Rui-Juan Xu, Bin Wang, Wei-Hong Ge

**Affiliations:** ^1^Department of Pharmacy, Nanjing Drum Tower Hospital, The Affiliated Hospital of Nanjing University Medical School, Nanjing, China; ^2^School of Basic Medicine and Clinical Pharmacy, China Pharmaceutical University, Nanjing, China; ^3^School of Biomedical Engineering, Faculty of Engineering and IT, University of Technology Sydney, Ultimo, NSW, Australia; ^4^Department of Sports Medicine and Adult Reconstruction Surgery, Nanjing Drum Tower Hospital, The Affiliated Hospital of Nanjing University Medical School, Nanjing, China; ^5^Department of Orthopaedic, Second Hospital of Shanxi Medical University, Taiyuan, China

## Abstract

This study was performed to assess the efficacy and safety of a topical diclofenac solution in patients with knee osteoarthritis (OA). PubMed, Embase, Cochrane Library, Web of Science, and Scopus databases were searched for randomized controlled trials until June 2020. The WOMAC pain, stiffness, physical function subscales, pain on walking, and the occurrence of adverse events were pooled to comprehensively analyse the efficacy and safety of topical diclofenac solution. All statistical analyses were conducted using Review Manager 5.3 software. Five RCTs were included, which provided high-quality evidence. In comparison to the vehicle control, the mean differences for WOMAC pain, stiffness, and physical function subscales, as well as pain on walking, were all statistically significant in favor of topical diclofenac solution. The safety of topical diclofenac solution was similar to the vehicle control, apart from adverse events involving application-site skin reactions. Topical diclofenac solution is effective and safe for use in patients with knee OA, but may cause minor skin reactions.

## 1. Introduction

Osteoarthritis (OA) is a highly prevalent degenerative joint disease, which can cause chronic pain and disability that significantly affect quality of life and the ability to perform daily activities. It has been reported that one in eight adults has osteoarthritis above 65 years of age [1]. Chronic OA management creates severe burdens for global healthcare systems and negative impacts on work productivity [2–5]. Depending on the stage of disease, treatment may range from conservative approaches to surgery for removing the joint. Current conservative management approaches for knee OA include extracorporeal shock wave [6], chondroitin sulfate [7], hyaluronic acid [8], and nonsteroidal anti-inflammatory drugs (NSAIDs) [9, 10]. Various complementary treatments have also been tested including acupuncture [11], Baduanjin exercises [12, 13], and herbal medicines [14, 15].

Among these conservative therapies, topical NSAIDs are strongly recommended for knee OA patients (level 1A) with no comorbidities, which have shown modest benefits over a course of 12 weeks according to high-quality evidence [16]. Also, topical NSAIDs are also strongly recommended for knee OA patients with gastrointestinal or cardiovascular comorbidities, as well as for patients with frailty. Topical diclofenac solution is one of the commonly used topical NSAIDs, which is a cutaneous solution (1.5% *w*/*w* or 2% *w*/*w* diclofenac sodium) indicated for the symptomatic relief of pain associated with knee OA, and is currently approved in Canada and several European countries. The solution base contains dimethyl sulfoxide (DMSO) to enhance the absorption of diclofenac sodium [17–19], which is applied directly to the affected knee.

In the current literature, one systematic review of randomized controlled trials (RCTs) had evaluated the efficacy and safety of topical diclofenac solution when used to treat knee OA [20], but did not include the more recent RCTs [21, 22]. A network meta-analysis also compared the relative efficacy and safety of topical diclofenac solution with another 11 topical NSAIDs [23], but this study did not include the outcomes on Western Ontario and McMaster Universities Arthritis Index (WOMAC) subscales and pain on walking. Recently, a study on individual patient data meta-analysis of RCTs showed that topical NSAIDs were effective for OA pain relief, and that people with higher OA pain at baseline experienced greater overall reductions in pain, although this may have been attributed to contextual or nonspecific, rather than specific, treatment effects [24]. The purpose of this study was to perform a meta-analysis using the currently available evidence from RCTs to investigate the efficacy and safety of using topical diclofenac solution in patients with knee OA.

## 2. Materials and Methods

### 2.1. Literature Search and Selection

Systematic literature searches were performed using the PubMed, Embase, Cochrane Library, Web of Science, and Scopus databases by two independent researchers (T.L. and B.W.). Publications were searched from January 1966 to June 2020, and studies were limited to RCTs in patients with knee OA. The search strategy included key search terms: (“Nonsteroidal Anti-Inflammatory Agents” or “NSAIDs” or “diclofenac” or “Pennsaid”) and (“Topical Administrations” or “Topical”) and (“Osteoarthritis” or “Degenerative Arthritis” or “OA”). The search filters were applied on “randomized controlled trial” or “RCT.” We used Google Scholar to increase the ability to identify all literature related to the topic of interest adequately. A systematic literature review was conducted by browsing abstracts of major conferences to identify the additional unpublished studies. In addition, the reference lists of previously published randomized trials, review articles, and meta-analysis were manually searched for additional eligible studies. Related articles and reference lists were searched to avoid misses.

All citations were downloaded into Endnote X9.1 (Clarivate Analytics). Duplicate records were removed electronically and manually. Two authors (Tao Ling and Bin Wang) screened the remaining articles at the title and abstract level followed by full text. In addition, the reference list of relevant systematic reviews and meta-analysis was scanned to identify potentially eligible studies. Citations were exported to Endnote, and duplicates were removed before the titles and abstracts, and the full text of remaining studies was then assessed according to the inclusion and exclusion criteria. The study protocol was registered with PROSPERO, number CRD42020186646.

### 2.2. Inclusion and Exclusion Criteria

The inclusion criteria for this meta-analysis: (1) studies involving patients with knee OA; (2) RCTs; (3) interventions were topical diclofenac solution and vehicle-placebo; (4) studies reporting outcomes on WOMAC and adverse events; and (5) studies published in English. The exclusion criteria for this meta-analysis: (1) secondary analyses, including pooled analyses; (2) study duration of less than 2 weeks; (3) studies involving multiple pharmacological interventions; and (4) unavailable full text.

### 2.3. Study Quality Assessment

The WOMAC LK3.0 osteoarthritis index was used, and pain on walking was analyzed as a separate efficacy variable [25, 26]. The Cochrane risk of bias assessment tool was used to determine the methodological quality of included RCTs [27]. A total of six domains were evaluated: random sequence generation, allocation concealment, participant blinding, outcome assessor blinding, incomplete outcome data, and selective reporting. Each domain was assigned a judgment of low risk of bias, high risk of bias, or unclear risk of bias. The judgments for each domain were made by strictly following the Cochrane Handbook V.5.1.0, Chapter 8.5.

### 2.4. Statistical Analysis

For the assessment of efficacy, the primary variable was changed in the WOMAC subscale score for pain. The secondary variables were changes in the WOMAC subscale scores for physical function and stiffness, as well as the pain on walking. All changes resulted from comparisons between baseline values and final assessment. A meta-analysis was conducted to compare the efficacy and safety of topical diclofenac solution with vehicle control. The heterogeneity of the effect size across the included studies was tested using the *Q* statistic (*P* < 0.05 was considered heterogeneous) and *I*^2^ statistic (*I*^2^ > 50% was considered heterogeneous). If there was no significant heterogeneity between studies, a fixed-effects model was used; otherwise, a random-effects model was used. Publication bias was assessed using visual inspection of funnel plots, where an asymmetrical funnel plot indicated potential publication bias [28]. We assessed funnel plot asymmetry using Bgger's tests, and defined significant publication bias as a *P* value < 0·05. All statistical analyses were conducted using Review Manager 5.3 software (RevMan 5.3, Cochrane Collaboration, Oxford, UK).

## 3. Results

### 3.1. Study Selection and Characteristics of Included Studies

**Figure 1:**
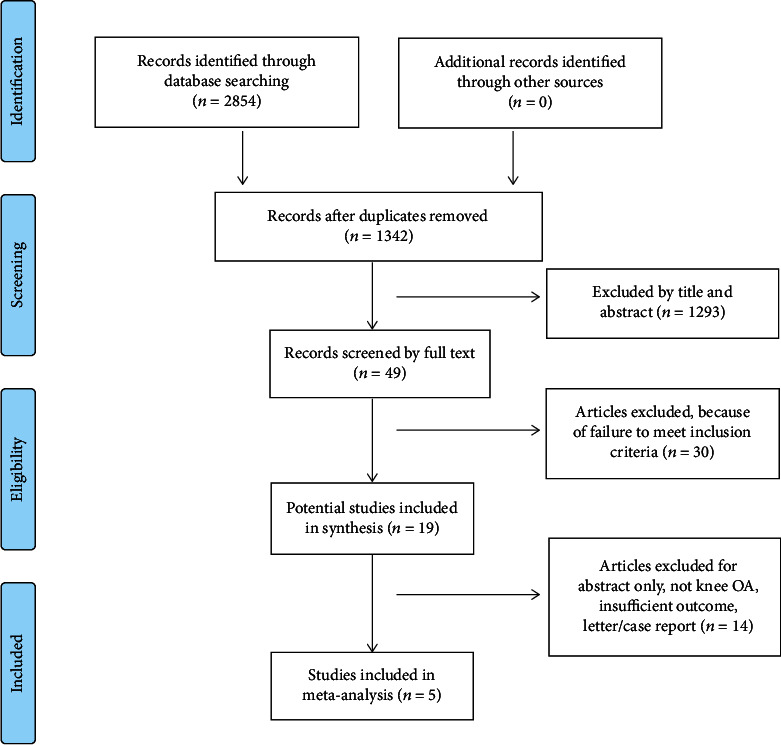
Flow diagram for the identification of included studies.

**Figure 2:**
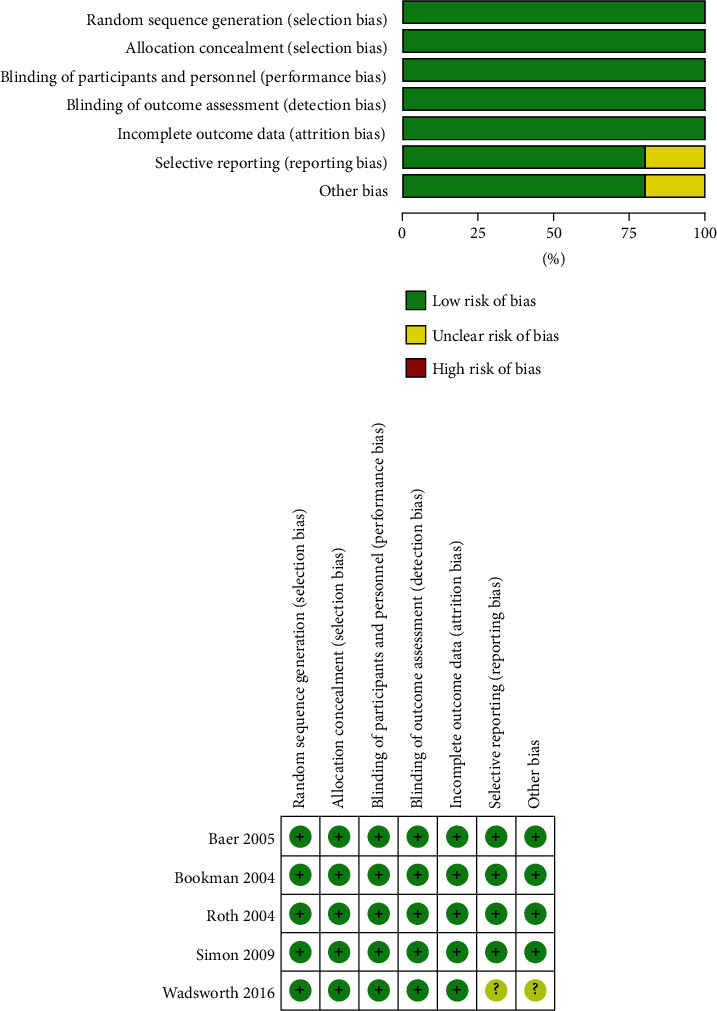
Risk of bias assessment for the included studies: (a) “Risk of bias” summary: review authors' judgments about each risk of bias item for each included study (b) “Risk of bias” graph: review authors' judgments about each risk of bias item presented as percentages across all included studies.

Of the 2073 studies identified from database searches, five trials [21, 22, 29–31] involving 1271 knee OA patients were included for data analysis. The characteristics and adverse events reported in the included RCTs were presented in Table 1. The experimental group was a topical diclofenac solution, and the control group was vehicle control. The average age of patients was 62.84 ± 10.35 (mean ± standard deviation) years, and 63.63% of patients were women. All trials were conducted in USA or Canada, and the mean trial duration was 7.6 weeks. The selection process for included studies was shown in a flow diagram (Figure 1). The risk of bias assessment showed that with the exception of one study [22], all included studies had a low risk of bias for all of the assessment criteria (Figure 2). The overall quality of the included studies was high.

### 3.2. Efficacy of Topical Diclofenac Solution

**Figure 3:**
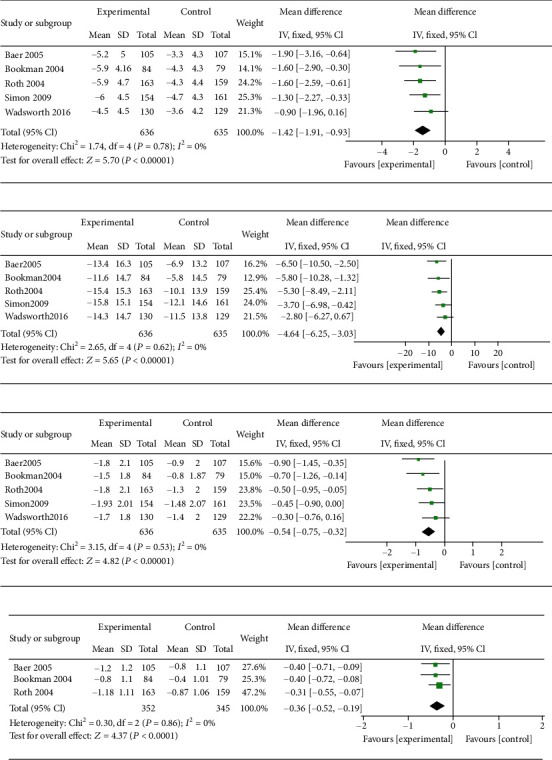
Meta-analysis of the efficacy of topical diclofenac solution compared to vehicle control. (a) WOMAC pain subscale, (b) WOMAC physical function subscale, (c) WOMAC stiffness subscale, and (d) pain on walking.

**Figure 4:**
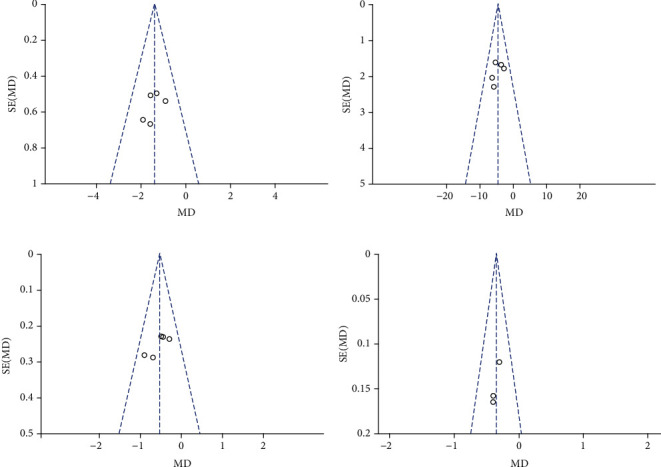
Funnel plots of the efficacy of topical diclofenac solution compared to vehicle control. (a) WOMAC pain subscale, (b) WOMAC physical function subscale, (c) WOMAC stiffness subscale, and (d) pain on walking.

A complete efficacy profile analysis including the WOMAC pain subscale, the WOMAC physical function subscale, the WOMAC stiffness subscale, and pain on walking was performed for all trials selected for inclusion in this meta-analysis (Figure 3). Topical diclofenac solution showed significant differences compared to vehicle control, where results are shown by mean differences (MD) and confidence interval (CI): WOMAC pain subscale (MD = −1.42; 95% CI -1.91 to -0.93; *P* < 0.0001), WOMAC physical function subscale (MD = −4.64; 95% CI -6.25 to -3.03; *P* < 0.0001), WOMAC stiffness subscale (MD = −0.54; 95% CI -0.75 to -0.32; *P* < 0.0001), and pain on walking (MD = −0.36; 95% CI -0.52 to -0.20; *P* < 0.0001). Results of the WOMAC pain, physical function and stiffness subscales, and the pain on walking showed no heterogeneity: (*I*^2^ = 0%; *P* = 0.78), (*I*^2^ = 0%; *P* = 0.62), (*I*^2^ = 0%; *P* = 0.53), and (*I*^2^ = 0%; *P* = 0.86), respectively. Funnel plots of the efficacy of topical diclofenac solution were shown in Figure 4. *P* values from Bgger's test indicated that there was no significant publication bias for the WOMAC pain, stiffness, physical function subscales, or pain on walking (*P* = 1.000, *P* = 0.806, *P* = 0.806, and *P* = 0.296, respectively).

### 3.3. Safety of Topical Diclofenac Solution

**Figure 5:**
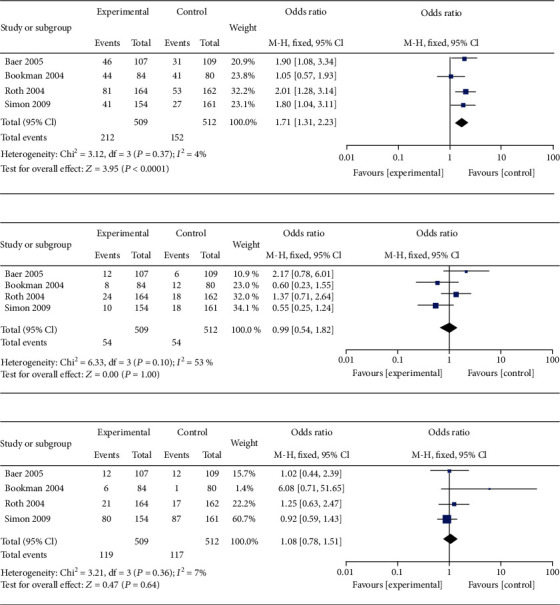
Meta-analysis of the safety of topical diclofenac solution compared to vehicle control (a) adverse events relating to application-site skin reactions, (b) adverse events relating to gastrointestinal tract reactions, and (c) adverse events relating to other reactions.

**Figure 6:**
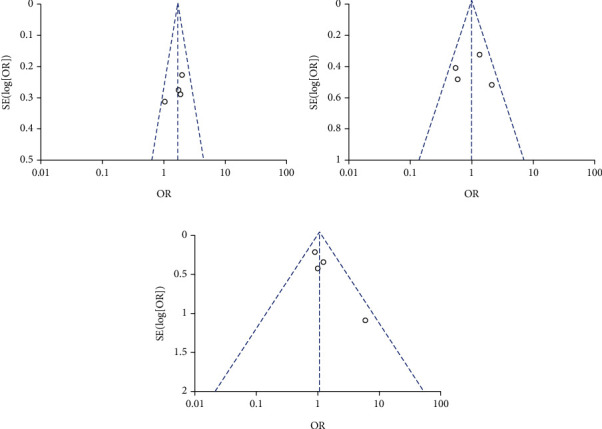
Funnel plots of the safety of topical diclofenac solution compared to vehicle control. (a) Adverse events relating to application-site skin reactions, (b) adverse events relating to gastrointestinal tract reactions, and (c) adverse events relating to other reactions.

**Figure 7:**
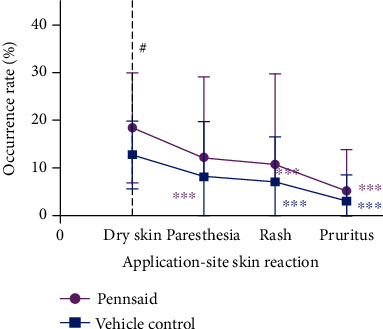
Occurrence rates of specific adverse events relating to application-site skin reactions ^#^*P* < 0.05 compared to vehicle control; ^∗∗∗^*P* < 0.001 compared to dry skin.

The meta-analysis of the safety of topical diclofenac solution compared to vehicle control is presented (Figure 5). There was a statistically significant difference in the occurrence of adverse events relating to application-site skin reactions between the topical diclofenac solution and vehicle control groups (OR = 1.71; 95% CI 1.31 to 2.23; *P* = 0.0001), and no heterogeneity was identified (*I*^2^ = 4%; *P* = 0.37). There were no statistical differences in the occurrence of adverse events relating to gastrointestinal tract reactions (OR = 0.99; 95% CI 0.54 to 1.82; *P* = 0.97) or other reactions such as asthma and dizziness (OR = 1.08; 95% CI 0.78 to 1.51; *P* = 0.72), with moderate (*I*^2^ = 53%; *P* = 0.10) and no (*I*^2^ = 7%; *P* = 0.36) heterogeneity identified in these analyses, respectively. Funnel plots of the safety of topical diclofenac solution were shown in Figure 6. *P* values from Bgger's test indicated that there was no significant publication bias for the occurrence of adverse events relating to application-site skin, gastrointestinal tract, or other reactions (*P* = 0.308, *P* = 0.734, and *P* = 0.308, respectively). Pooled analysis of specific adverse events relating to application-site skin reactions showed that dry skin had significantly higher occurrence rates for topical diclofenac solution compared to vehicle control, while paresthesia, rash, and pruritus had similar occurrence rates in both groups (Figure 7). Dry skin was by far the most common type of application-site skin reaction in both groups compared to other types of adverse reactions.

## 4. Discussion

Topical administration of medication is often preferred in clinical practice due to the advantages of having a high local drug concentration, good treatment effect, and convenient application and has been recommended for use in the treatment of knee OA [16, 32, 33]. For diclofenac sodium, topical administration can avoid systemic exposure resulting from oral medication, which reduces the occurrence rate of adverse events [34–39]. This is particularly beneficial as it reduces the risks associated with polypharmacy for OA patients, the majority of whom are elderly individuals and may have other comorbidities requiring oral medication. Our meta-analysis of five RCTs comparing topical administration of topical diclofenac solution and vehicle control for the treatment of knee OA showed that topical diclofenac solution was significantly more effective at symptom relief according to the 3 WOMAC subscale scores and pain on walking. The safety of topical diclofenac solution was comparable to the vehicle control with a similar occurrence rate of adverse events, except for application-site skin reactions which were significantly higher in the topical diclofenac solution group. Dry skin was the most common type of reaction in the topical diclofenac solution group, which also occurred at a much higher rate than in the vehicle control group. These findings are consistent with an earlier meta-analysis published in 2006 [20], although this earlier analysis did not include the more recent topical diclofenac solution trials [21, 22], one of which was the only RCT on 2% *w*/*w* topical diclofenac solution [22].

Other studies have compared topical diclofenac solution with oral diclofenac in the symptomatic treatment of knee OA. In an equivalence study, topical diclofenac solution was shown to provide symptomatic relief to the same extent as oral diclofenac, with increased occurrence of minor local skin irritation (27%) but significantly reduced incidence of severe gastrointestinal adverse events and abnormal values in liver function tests [40]. Similar results were obtained in another study, where topical diclofenac was shown to have a higher incidence of dry skin (18.2%) but fewer digestive system and laboratory abnormalities [21]. The study concluded that topical diclofenac in DMSO was an effective treatment option for knee OA with similar efficacy but improved tolerability compared to oral diclofenac. In general, 1.5% topical diclofenac solution (19.3 mg/40 drops, twice daily) and 2% *w*/*w* topical diclofenac solution (40.4 mg/2 mL, twice daily) provide a similar daily dose to oral diclofenac (75 mg, twice daily), with equivalent efficacy but significantly lower exposure and hence fewer adverse events [21, 37, 40].

A few studies have compared topical diclofenac solution with 1% *w*/*w* diclofenac sodium gel for treating knee OA. A comparative subjective assessment study showed that 1.5% *w*/*w* topical diclofenac solution had a number of characteristics that were rated significantly better than 1% *w*/*w* diclofenac sodium gel, such as “odor/smell” and “stickiness/tackiness on knee,” and more patients preferred or highly preferred topical diclofenac solution over 1% *w*/*w* diclofenac sodium gel [41]. A network meta-analysis of topical NSAIDs for OA treatment showed that diclofenac solution and diclofenac gel had similar effects on pain relief and functional improvement in RCTs compared to placebo [23]. The risk of skin adverse effects was higher for diclofenac solution, but the risk of gastrointestinal adverse effects and withdrawal due to adverse effects were higher for diclofenac gel.

Since topical diclofenac solution is a cutaneous solution, its indication for symptomatic pain relief in knee OA is based on the ability for diclofenac sodium to be absorbed through the skin, which is enhanced by the presence of DMSO. Topical diclofenac solution is suitable for application to the knee joint due to the anatomical joint structure bounded by thin tissue layers. Although topical diclofenac solution can be theoretically applied to relieve osteoarthritic pain in large, deep joints covered by layers of muscle or other soft tissues, such as the hip or spine, no data are currently available. High-quality RCTs will need to be performed to evaluate the efficacy of topical diclofenac solution in such applications, which would depend on the efficiency of absorption into the joint.

This meta-analysis has some limitations that should be taken into consideration when interpreting the findings. First, some of the included RCTs had limitations associated with the reported outcomes. For instance, day 1 efficacy scores may have been used instead of baseline scores for some patients. Two of the trials had a duration of 4 weeks, which may not allow adequate assessment of potential long-term safety concerns, such as gastrointestinal adverse effects. Second, there were limitations relating to the characteristics of the included studies. Only one of the five included RCTs used a 2% topical diclofenac solution while the other four used 1.5% *w*/*w* topical diclofenac solution, which limits the ability to generalize the findings in this meta-analysis for all topical diclofenac solution formulations. Furthermore, all of the included studies were sponsored by the manufacturers of topical diclofenac solution. Although all of these studies generally had a low risk of bias, the motivation for conducting and publishing these studies should be considered. Finally, there may be high-quality non-English studies that could have influenced the outcomes of our meta-analysis, but were excluded due to the selection criteria.

## 5. Conclusion

This meta-analysis showed that topical diclofenac solution provided effective treatment for patients with knee OA and achieved significantly better results compared to vehicle control when assessed by the WOMAC subscales for pain, physical function, and stiffness, as well as pain on walking. The main adverse events during treatment were application-site reactions, particularly dry skin. In summary, the available evidence on the combined efficacy and safety of topical diclofenac solution makes it a viable treatment option for symptomatic relief in knee OA.

## Figures and Tables

**Table 1:** Characteristics and adverse events reported in the included RCTs.

Study	Pennsaid® therapy	Age (mean (SD))	Sample (F%)	Areas	Duration (W)	Adverse events (no. (and %) of patients)		
						Application-site skin	Gastrointestinal	Other reaction
Bookman et al. 2004 [29]	Diclofenac solution, 1.5% (40 drops), 4 times daily	G1: 62.5 (11.7) G2: 62.1 (11.4)	G1: 84 (62) G2: 80 (68)	Canada	4	G1: 44 (52.38) G2: 41 (51.25)	G1: 8 (9.52) G2: 12 (15.00)	G1: 6 (7.14) G2: 1 (1.25)
Roth and Shainhouse 2004 [30]	Diclofenac solution, 1.5% (40 drops), 4 times daily	G1: 63.4 (10.5) G2: 64.9 (10.6)	G1: 164 (68.9) G2: 162 (66.7)	USA	12	G1: 81 (49.39) G2: 53 (32.72)	G1: 24 (14.63) G2: 18 (11.11)	G1: 21 (12.8) G2: 17 (10.49)
Baer et al. 2005 [31]	Diclofenac solution, 1.5% (40 drops), 4 times daily	G1: 65.0 (11.0) G2: 64.6 (10.9)	G1: 107 (52.3) G2: 109 (60.6)	Canada	6	NR	G1: 12 (11.21) G2: 6 (5.50)	G1: 12 (11.21) G2: 12 (11.01)
Simon et al. 2009 [21]	Diclofenac solution, 1.5% (40 drops), 4 times daily	G1: 61.7 (9.8) G2: 62.1 (9.3)	G1: 154 (67.5) G2: 161 (55.9)	Canada	12	G1: 41 (26.62) G2: 27 (16.77)	G1: 10 (6.49) G2: 18 (11.18)	G1: 80 (51.95) G2: 87 (54.04)
Wadsworth et al. 2016 [22]	Diclofenac solution, 2% (2 mL), 4 times daily	G1: 60.2 (9.2) G2: 61.9 (9.1)	G1: 130 (64.6) G2: 129 (69.8)	USA	4	G1: 43 (33.08) G2: 75 (58.14)	NR	NR

G1: topical diclofenac solution group; G2: vehicle control group; F: female; W: weeks; NR: not reported.

## Data Availability

The data used to support the findings of this study are included within the article.
